# Circulating microRNAs in the early prediction of disease recurrence in primary breast cancer

**DOI:** 10.1186/s13058-018-1001-3

**Published:** 2018-07-11

**Authors:** Chara Papadaki, Michalis Stratigos, Georgios Markakis, Maria Spiliotaki, Georgios Mastrostamatis, Christoforos Nikolaou, Dimitrios Mavroudis, Sofia Agelaki

**Affiliations:** 1 Laboratory of Translational Oncology, School of Medicine, University of Crete, Heraklion, 71003 Heraklion, Crete Greece; 2 Department of Medical Oncology, University General Hospital of Heraklion, 1352 PO BOX, 711 10 Heraklion, Crete Greece; 3 Department of Agricultural, Technological Education Institute of Heraklion, 72100 Heraklion, Crete Greece; 4 Computational Genomics Group, Department of Biology, University of Crete, 70013 Heraklion, Greece; 5 Institute of Molecular Biology and Biotechnology, Foundation for Research and Technology, 70013 Heraklion, Crete Greece

**Keywords:** Circulating miRNAs, Breast cancer, Relapse, Metastasis, Dormancy

## Abstract

**Background:**

In primary breast cancer metastases frequently arise from a state of dormancy that may persist for extended periods of time. We investigated the efficacy of plasma micro-RNA (miR)-21, miR-23b, miR-190, miR-200b and miR-200c, related to dormancy and metastasis, to predict the outcome of patients with early breast cancer.

**Methods:**

miRNAs were evaluated by RT-qPCR in plasma obtained before adjuvant chemotherapy. miRNA expression, classified as high or low according to median values, correlated with relapse and survival. Receiver operating characteristic (ROC) curves were constructed to determine miRNA sensitivity and specificity.

**Results:**

miR-21 (*p* < 0.001), miR-23b (*p* = 0.028) and miR-200c (*p* < 0.001) expression were higher and miR-190 was lower (*p* = 0.013) in relapsed (*n* = 49), compared to non-relapsed patients (*n* = 84). Interestingly, miR-190 was lower (*p* = 0.0032) in patients with early relapse (at < 3 years; *n* = 23) compared to those without early relapse (*n* = 110). On the other hand, miR-21 and miR-200c were higher (*p* = 0.015 and *p* < 0.001, respectively) in patients with late relapse (relapse at ≥ 5 years; *n* = 20) as compared to non-relapsed patients. High miR-200c was associated with shorter disease-free survival (DFS) (*p* = 0.005) and high miR-21 with both shorter DFS and overall survival (OS) (*p* < 0.001 and *p* = 0.033, respectively) compared to low expression. ROC curve analysis revealed that miR-21, miR-23b, miR-190 and miR-200c discriminated relapsed from non-relapsed patients. A combination of of miR-21, miR-23b and miR-190 showed higher sensitivity and specificity in ROC analyses compared to each miRNA alone; accuracy was further improved by adding lymph node infiltration and tumor grade to the panel of three miRs (AUC 0.873). Furthermore, the combination of miR-200c, lymph node infiltration, tumor grade and estrogen receptor predicted late relapse (AUC 0.890).

**Conclusions:**

Circulating miRNAs are differentially expressed among relapsed and non-relapsed patients with early breast cancer and predict recurrence many years before its clinical detection. Our results suggest that miRNAs represent potential circulating biomarkers in early breast cancer.

**Electronic supplementary material:**

The online version of this article (10.1186/s13058-018-1001-3) contains supplementary material, which is available to authorized users.

## Background

Despite significant advances in diagnosis and treatment of early breast cancer, almost 30% of patients will eventually have local or distant recurrence [1–3]. Recurrence is considered to result from cancer cells that persist after surgery and systemic therapy and remain in a dormant state for many years before they start proliferating and form local or distant metastases [4, 5]. Strategies to improve the management of patients with early disease should include the development of novel biomarkers for the early recognition of patients at high risk of relapse.

Clinicopathological parameters are commonly used for the prediction of patients’ prognosis; however, they often lack individualized validity for the identification of patients at high risk, due to significant inter-patient heterogeneity [6]. In addition, molecular profiling tests have been developed for prognostication but their routine clinical implementation is problematic [7]. Furthermore, the genetic profiling of solid tumors is currently performed on biopsies that might fail to reflect intra-tumoral heterogeneity and limit the opportunity to track genetic alterations occurring during cancer evolution [8]. Therefore, there is an unmet need to identify novel non-invasive biomarkers for the better prediction of the risk of recurrence in breast cancer.

MicroRNAs (miRNAs), a large family of small (20–22 nucleotides) non-coding RNAs, regulate approximately 30% of the genes in the human genome at the post-transcriptional level, by binding to the complementary sequences of the 3′- untranslated region (3’-UTR) of their target messenger RNAs (mRNAs), leading to either mRNA degradation or inhibition of protein translation [9]. miRNAs are deregulated in cancer, acting as both oncogenes and tumor suppressor genes [10]. The altered expression of miRNAs has been associated with poor clinical outcome in patients diagnosed with a variety of tumors [11]. In the past decade miRNAs have emerged as promising biomarkers in breast cancer and have been increasingly identified in biological fluids such as serum or plasma as circulating miRNAs [12]. Circulating miRNAs are significantly stable in biological fluids [13, 14] and could potentially serve as a “liquid biopsy” for the real-time evaluation of tumor status.

The assessment of dormancy and metastasis-related miRNAs could be of importance for the identification of patients at high risk of relapse. The mechanisms that lead to dormancy or enable the formation of metastases remain poorly understood. Data from in vitro models or expression analysis in patients with breast cancer suggest that miR-21, miR-23b, miR-190 and the miR-200 family members, such as miR-200b and miR-200c, are important in cancer dormancy and metastasis. An epithelial to mesenchymal transition (EMT)-related gene signature in the primary tumor has been associated with both stromal activation and escape from dormancy in breast cancer [15], suggesting that intrinsic EMT features may regulate the transition of disseminated tumor cells into a dormant phenotype with the ability to outgrow as recurrent disease. In another report, the activation of the EMT program, as orchestrated by the key regulator of EMT, Zeb1, was sufficient to promote escape from latency and stimulate the development of metastases [16]. The miR-200 family regulates EMT by targeting the ZEB1/2-E-cadherin axis [17], whereas in other studies, elevated levels of miR-200 family have induced EMT and promoted metastasis in breast cancer [18]. Several lines of evidence suggest that miR-21 is oncogenic in various types of cancer by suppressing several apoptotic and tumor suppressor genes [19] and by inducing cell proliferation, migration, invasion and metastasis. miR-23b has been shown to promote tumor dormancy in the metastatic niche [20], whereas miR-190 upregulation has been associated with prolonged tumor dormancy in fast-growing tumors such as osteosarcomas and glioblastomas [21].

Based on the above, the aim of the present study was to investigate the expression of miR-21, miR-23b, miR-190, miR-200b and miR-200c in the plasma of patients with early breast cancer and evaluate their role in the prediction of patients’ outcomes.

## Methods

### Patients’ characteristics and sample collection

**Fig. 1:**
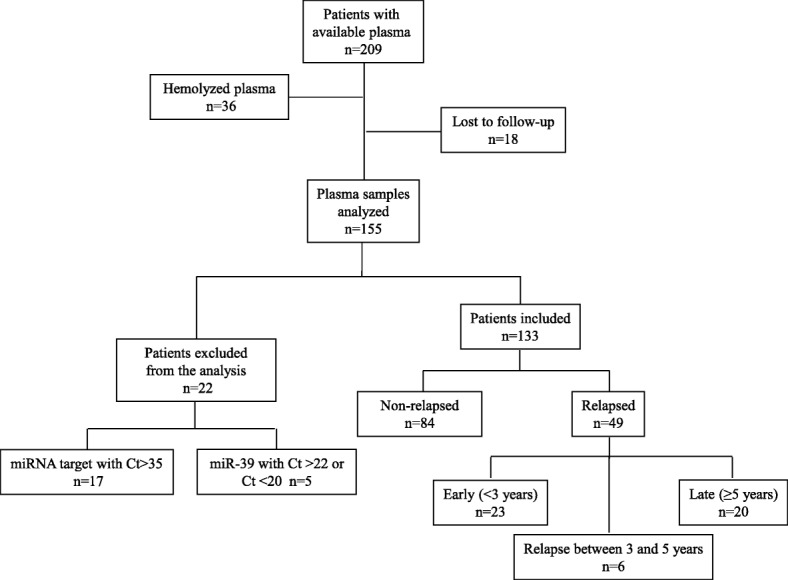
Flow chart of the study. Ct, cycle threshold

A total of 209 consecutive patients with early breast cancer who underwent surgery followed by adjuvant chemotherapy administered at the Department of Medical Oncology of the University Hospital of Heraklion (Crete, Greece) between years 2003 and 2010 and had available plasma samples, were included in the present study. Plasma samples were obtained after the surgical resection of the primary tumor and before the initiation of adjuvant chemotherapy. Plasma samples were also collected from 23 normal blood donors to serve as controls. All patients and normal donors had provided signed informed consent to participate in the study, which was approved by the Ethics and Scientific Committee of Department of Medical Oncology of the University Hospital of Heraklion (ID 13998/8–10-2104; Crete, Greece). Clinical characteristics and follow-up information for each patient were prospectively collected. Peripheral blood from healthy donors and patients was drawn early in the morning and was collected in EDTA tubes. Plasma was subsequently isolated within 2 h by centrifugation at 2500 rpm for 15 min at 4 °C, followed by a second centrifugation at 2000 g for 15 min at 4 °C to remove cellular debris. Samples were kept in aliquots at ˗ 80 °C until further use. Plasma samples presenting a change in color to pink, suggesting the presence of hemolysis, were not processed for further analysis (Fig. 1).

### RNA isolation

Plasma samples were thawed on ice and centrifuged at 10000 rpm for 10 min in order to remove cellular debris. Total RNA was extracted from 400 μl of plasma using Trizol LS (Ambion, Life Technologies). After denaturation, 5 μl containing 25 fmoles of a synthetic *Caenorhabditis elegans* miRNA cel-miR-39 (Qiagen Inc., USA) was added to each sample as an endogenous control to allow for normalization of sample-to-sample variation. Aqueous phase was separated from organic phase by adding 250 μl chloroform followed by incubation on ice for 10 min. After centrifugation, an equal volume of 700 μl of supernatant, from each sample, was transferred to an Eppendorf tube. Then, RNA was precipitated by adding 0.7 volumes of isopropanol and 1 μl glycogen followed by incubation at ˗ 80 °C, overnight. On the next day, and after centrifugation, RNA pellet was washed three times with 75% ethanol, air dried and finally resuspended in 50 μl RNAse-free water. RNA from all samples was kept in aliquots at ˗ 80 °C until further use in the subsequent real-time qPCR.

### Quantitative real-time PCR analysis and miRNA expression

Reverse transcription and RT-qPCR was performed according to manufacturer’s instructions and as previously described [13]. Total RNA input of 1.67 μl was reverse transcribed using the TaqMan miRNA Reverse Transcription kit and miRNA specific stem-loop primers (Applied Biosystmes, Foster City, CA, USA) in a 5-μl reaction comprising 1 mM dNTPs, 1 × PCR Reverse Transcription Buffer, 0.787 μl H_2_O, 3.3 units Multiscribe Reverse Transcriptase, 0.252 units RNase inhibitors and 0.2 × RT-specific stem-loop primers. The reaction was performed in a Peltier Thermal Cycler PTC-200 at 16 °C for 30 min, 42 °C for 30 min and 85 °C for 5 min. Complementary DNA (cDNA) was diluted at 30 μl and each miRNA was assessed by RT-qPCR in a 5-μl reaction comprising 1 × of TaqMan 2× Universal PCR Mater Mix, No AmpErase UNG, 0.25 μl of TaqMan miRNA Assay and 2.25 μl of diluted cDNA. The quantitative real- time PCR reaction was carried out at 95 °C for 10 min, followed by 40 cycles of 95 °C for 15 min and 60 °C for 1 min on a ViiA 7 Real-Time PCR System (Applied Biosystems, Foster City, CA, USA). All the assays were performed in triplicates. Appropriate negative controls were used in both cDNA synthesis and RT-qPCR reactions where RNA input was replaced by H_2_O and no template control was used, respectively. The average expression levels for each miRNA were calculated by the 2^-ΔCt^ method relative to the average of miR-23a.

Due to the lack of consensus concerning the normalization of circulating miRNAs we used miR-23a as a reference gene that was stably and reproducibly expressed among patients’ groups (Mann-Whitney test, *p* = 0.458) and among patients and normal donors (Mann-Whitney test, *p* = 0.12) [22]. Finally, the fold change in target miRNAs relative to miRNAs expressed in normal controls was calculated by the 2^-ΔΔCt^ method [23]. Samples with mean cycle threshold (Ct) > 35 for target miRNAs (*n* = 17) were excluded from the analysis. In addition, samples with mean Ct > 22 or Ct < 20 of cel-miR-39 (*n* = 5), suggesting RNA extraction was not efficient, were also excluded. Moreover, plasma samples were tested for contamination with red blood cells by measuring miR-451 and miR-23a expression levels [24]. Samples contaminated with red blood cells were not processed for further analysis.

### Statistical analysis

The statistical analysis was performed using the SPSS software package, version 22.0 (SPSS Inc. Chicago IL, USA). Cutoff points were set at the median value for expression of each miRNA. Patients with miRNA expression above or equal to the median values were characterized as having high expression, whereas patients with miRNA expression below the median were characterized as having low expression. Spearman’s test was used to test correlation between expression of the different miRNAs. The Mann-Whitney U-test and Kruskal Wallis test were used to estimate associations between miRNA expression and clinicopathological characteristics. Differences in clinicopathological characteristics between relapsed and non-relapsed patients were evaluated by Pearson’s chi-square test. The associations between circulating miRNA expression levels and disease-free survival (DFS) or overall survival (OS) were assessed by the Kaplan-Meier method, log rank test (Mantel-Cox) and Cox proportional hazard regression models. DFS was calculated from the date of surgery until the date of relapse or death from any cause, whereas OS was calculated from the date of surgery until the date of death from any cause or last follow up. The Mann-Whitney test was used to examine the differential expression between the different groups of patients. To evaluate the value of circulating miRNAs in predicting relapse, receiver operating characteristics (ROC) curves were constructed and area under the curve (AUC) calculated. The Youden index (sensitivity + specificity – 1) was used to set the optimal cutoff point. Logistic regression analyses were performed to identify the best discriminating combinations of miRNAs with clinicopathological features. Cross-validation analysis was implemented in R using a generalized linear model for logistic regression, with recurrence/non-relapse as binary target variables (http://www.r-project.org/). Statistical significance was set at *p* < 0.05 (two-sided test). This report is written according to the reporting recommendations for tumor marker prognostic studies (REMARK criteria) [25].

## Results

### Study design and patients’ characteristics

The flow diagram of the study and patients’ characteristics are summarized in Fig. 1 and Table 1, respectively. Plasma samples from 155 patients with early breast cancer and from 23 healthy women were processed for RNA extraction. There were 22 patients excluded from the analysis as described above. After a median follow-up period of 94.3 months (range 14.33–159.30), 84 out of the 133 patients with breast cancer who were included in the analysis remained disease-free and 49 had relapsed. Demographics and clinical characteristics were similar between patients who remained disease-free and those who developed recurrence, except for the proportions of patients with tumor size of > 5 cm (T3) and four or more infiltrated axillary lymph nodes, which were higher in patients who had recurrence (*p* = 0.015 and *p* = 0.003, respectively; Table 1). Patients were divided into three groups according to the clinical outcome: (i) patients who remained disease-free during the whole follow-up period (*n* = 84), (ii) patients with early relapse, defined as relapse within 3 years post-surgery (< 3 years; *n* = 23) and (iii) patients with late relapse, defined as relapse presenting at 5 years or more post-surgery (≥ 5 years; *n* = 20). Consequently, 6 out of 49 relapses were observed in between 3 and 5 years. Patients’ characteristics for the groups (ii) and (iii) are shown in Table 2. The median age was 52, 55 and 53 years in each group, respectively.

**Table 1:** Characteristics of patients with early breast cancer

	All patients		No relapse		Relapse		
Characteristic	Number	Percentage	Number	Percentage	Number	Percentage	*P*
Number of patients	133		84	63	49	37	
Age (years)							ns^a^
Median	54		52		56		
Range	27–79		35–79		27–75		
Menopausal status							ns^a^
Premenopausal	57	42,9	40	47.6	17	34.7	
Postmenopausal	76	57.1	44	52.4	32	65.3	
Tumor size (cm)							0.015^a^
T1	60	45.1	42	50	18	36.7	
T2	66	49.6	41	48.8	25	51	
T3	7	5,3	1	1.2	6	12.3	
Histological grade							ns^a^
I	6	4.5	6	7.1			
II	59	44.5	41	48.8	18	36.7	
III	58	43.6	31	36.9	27	55.1	
Lobular	5	3.7	3	3.6	2	4.1	
Unknown	5	3.7	3	3.6	2	4.1	
Infiltrated lymph nodes							0.001^a^
0	49	36.8	37	44.0	12	24.5	
1–3	44	33.1	28	33.3	16	32.7	
≥ 4	34	25.6	13	15.5	21	42.8	
Unknown	6	4.5	6	7.2			
ER status							
Positive	88	66.2	56	66.7	32	65.3	
Negative	43	32.3	26	31	17	34.7	ns^a^
Unknown	2	1.5	2	2.3			
PR status							ns^a^
Positive	90	67.7	61	72.6	29	59.2	
Negative	41	30.8	21	25	20	40.8	
Unknown	2	1.5	2	2.4			
HER2 status							ns^a^
Positive	15	11.3	7	8.3	8	16.3	
Negative	112	84.2	72	85.7	40	81.6	
Unknown	6	4.5	5	6	1	2.1	
Adjuvant chemotherapy							ns^a^
Anthracycline-based	13	9.8	9	10.7	4	8.2	
Taxanes+anthracyclines	90	67.7	51	60.7	39	79.6	
Taxane-based	21	15.8	15	17.9	6	12.2	
Others	9	6.7	9	10.7			
Hormone therapy							ns^a^
Yes	103	77.5	68	80.9	35	71.4	
No	28	21.0	14	16.7	14	28.6	
Unknown	2	1.5	2	2.4			

*ER* estrogen receptor, *PR* progesterone receptor, *HER2* human epidermal growth factor receptor 2, *ns* not significant

^a^Pearson’s chi-squared test for comparison between patients with relapse and without relapse

**Table 2:** Characteristics of patients with early (< 3 years) and late (≥ 5 years) relapse

	Early (< 3 years)		Late (≥ 5 years)		
Characteristic	Number	Percentage	Number	Percentage	*P*
Number of patients	23	17	20	15	
Age (years)					ns^a^
Median	55		53		
Range	27–75		38–74		
Menopausal status					ns^a^
Premenopausal	8	34.8	7	35	
Postmenopausal	15	65.2	13	65	
Tumor size (cm)					ns^a^
T1	7	30.4	8	40	
T2	12	52.2	10	50	
T3	4	17.4	2	10	
Histological grade					ns^a^
I	0		0		
II	10	43.5	6	30	
III	12	52.2	14	70	
Lobular	1	4.3			
Infiltrated lymph nodes					ns^a^
0	7	30.4	3	15	
1–3	9	39.2	6	30	
≥ 4	7	30.4	11	55	
ER					< 0.001^a^
Positive	9	39.2	19	95	
Negative	14	60.8	1	5	
PR					0.006^a^
Positive	10	43.5	17	85	
Negative	13	56.5	3	15	
Unknown					
HER2					ns^a^
Positive	4	17.4	4	20	
Negative	18	78.3	16	80	
Unknown	1	4.3			
Adjuvant chemotherapy					ns^a^
Anthracyclines-based	2	8.7	2	10	
Taxanes+anthracyclines	16	69.6	17	85	
Taxane-based	5	21.7	1	5	
Hormone therapy					0.002^a^
Yes	12	52.2	19	95	
No	11	47.8	1	5	

*ER* estrogen receptor, *PR* progesterone receptor, *HER2* human epidermal growth factor receptor 2, *ns* not significant

Pearson’s chi-squared test for comparison between patients with relapse and without relapse

### miRNA expression and statistical correlations

No significant associations were observed between miRNA expression (high expression, low expression) and age, menopausal status, tumor size, histological grade, number of infiltrated lymph nodes, estrogen receptor (ER), progesterone receptor (PR) or human epidermal growth factor receptor 2 (HER2) status (chi-square test, *p* > 0.05). However, miR-21 expression was higher in PR-negative as compared to PR-positive patients (63.4% vs 36.6%; chi-square test, *p* = 0.038). As expected, there was strong correlation between expression of miR-200b and miR-200c (Spearman’s Rho 0.628; *p* < 0.001) that belong to the same miR-200 family. Moreover, there was strong correlation between miR-21 and miR-200b (Spearman’s Rho 0.447; *p* < 0.001) and miR-200c (Spearman’s Rho 0.540; *p* < 0.001) expression, as well. Weaker but still significant association was observed between the dormancy-related miR-23b and miR-190 (Spearman’s Rho 0.236; *p* < 0.001) (Table 3).

**Table 3:** Coefficients of correlation among five miRNAs

	miR-21	miR-23b	miR-190	miR-200b	miR-200c
miR-21	1.000				
miR-23b	0.109	1.000			
miR-190	0.160	0.236*	1.000		
miR-200b	0.447**	−0.144	0.005	1.000	
miR-200c	0.540**	0.145	0.081	0.628**	1.000

**p < 0.01; *p < 0.05

### miRNA expression and clinical outcome

**Fig. 2:**
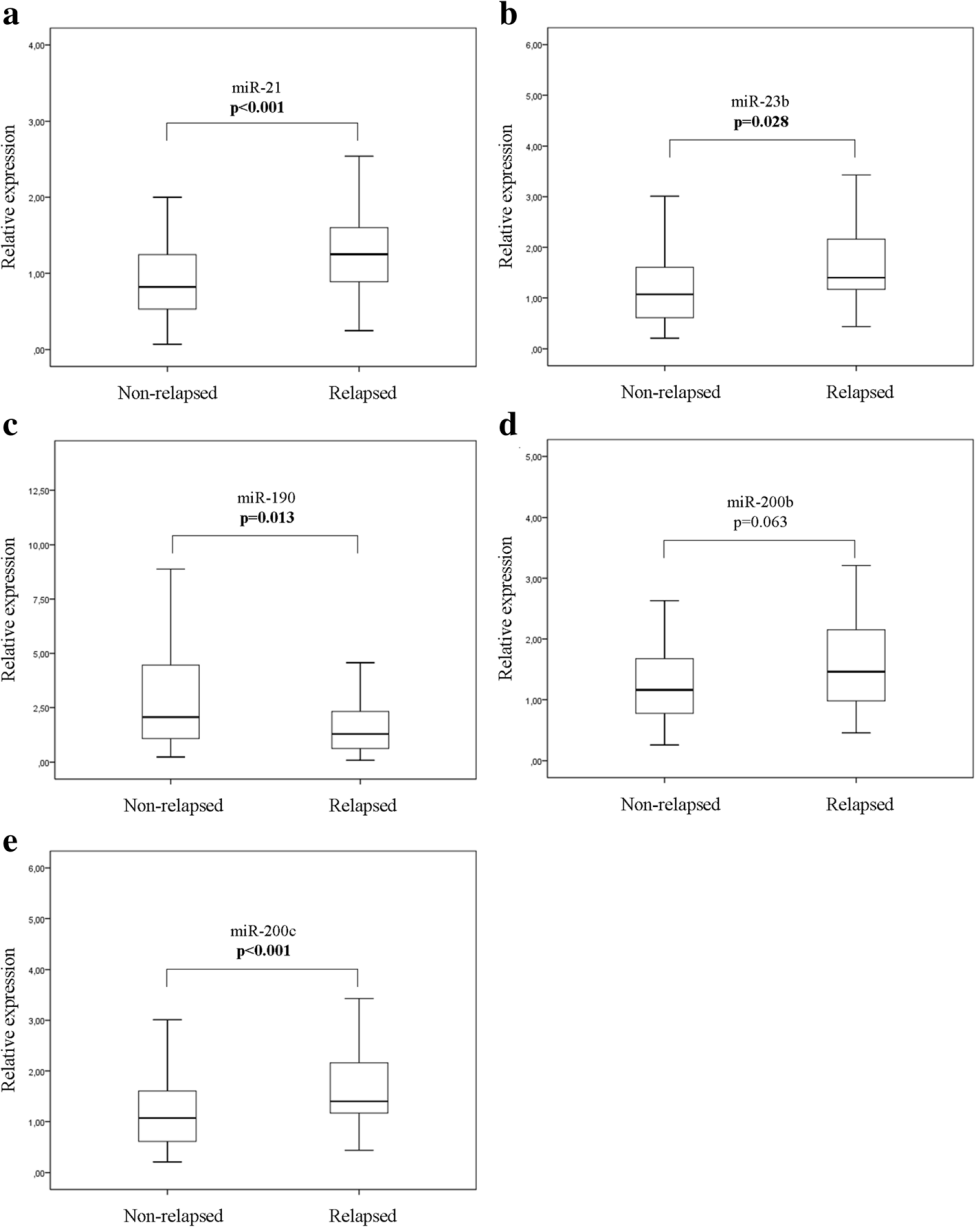
Relative expression levels of circulating miRNAs in relapsed and non-relapsed patients. Plasma levels of miR-21 (**a**), miR-23 (**b**), miR-190 (**c**), miR-200b (**d**) and miR-200c (**e**) were evaluated by RT-qPCR. Statistically significant differences were determined using the Mann-Whitney test. *P* values are shown

**Fig. 3:**
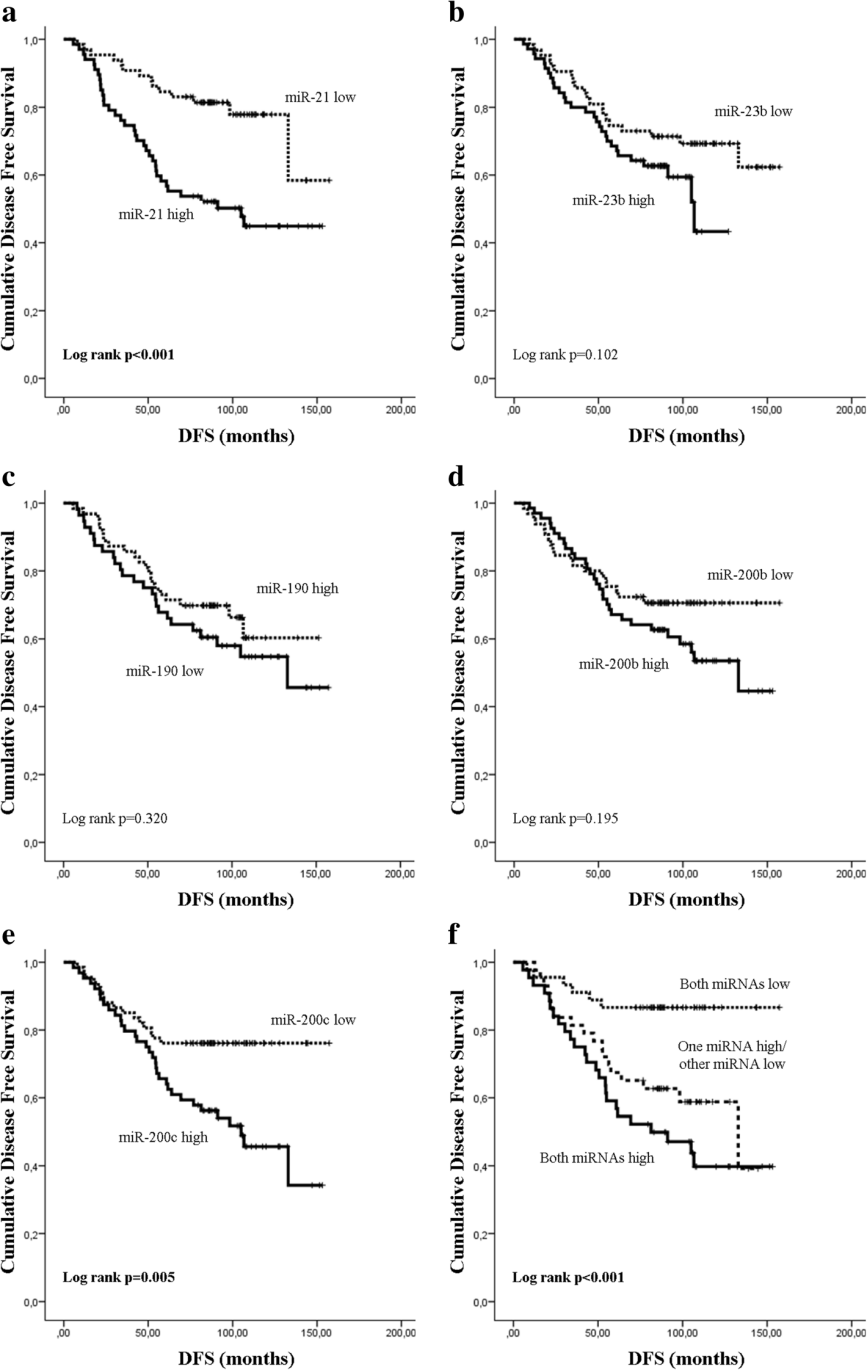
Kaplan-Meier analysis of disease-free survival (DFS) according to the expression of circulating miRNAs and their combination. DFS in patients with high or low expression of miR-21 (**a**), miR-23b (**b**), miR-190 (**c**), miR-200b (**d**), miR-200c (**e**) and the combination of miR-21 and miR-200c (**f**). Curves were compared using the log rank test. *P* values are shown

**Fig. 4:**
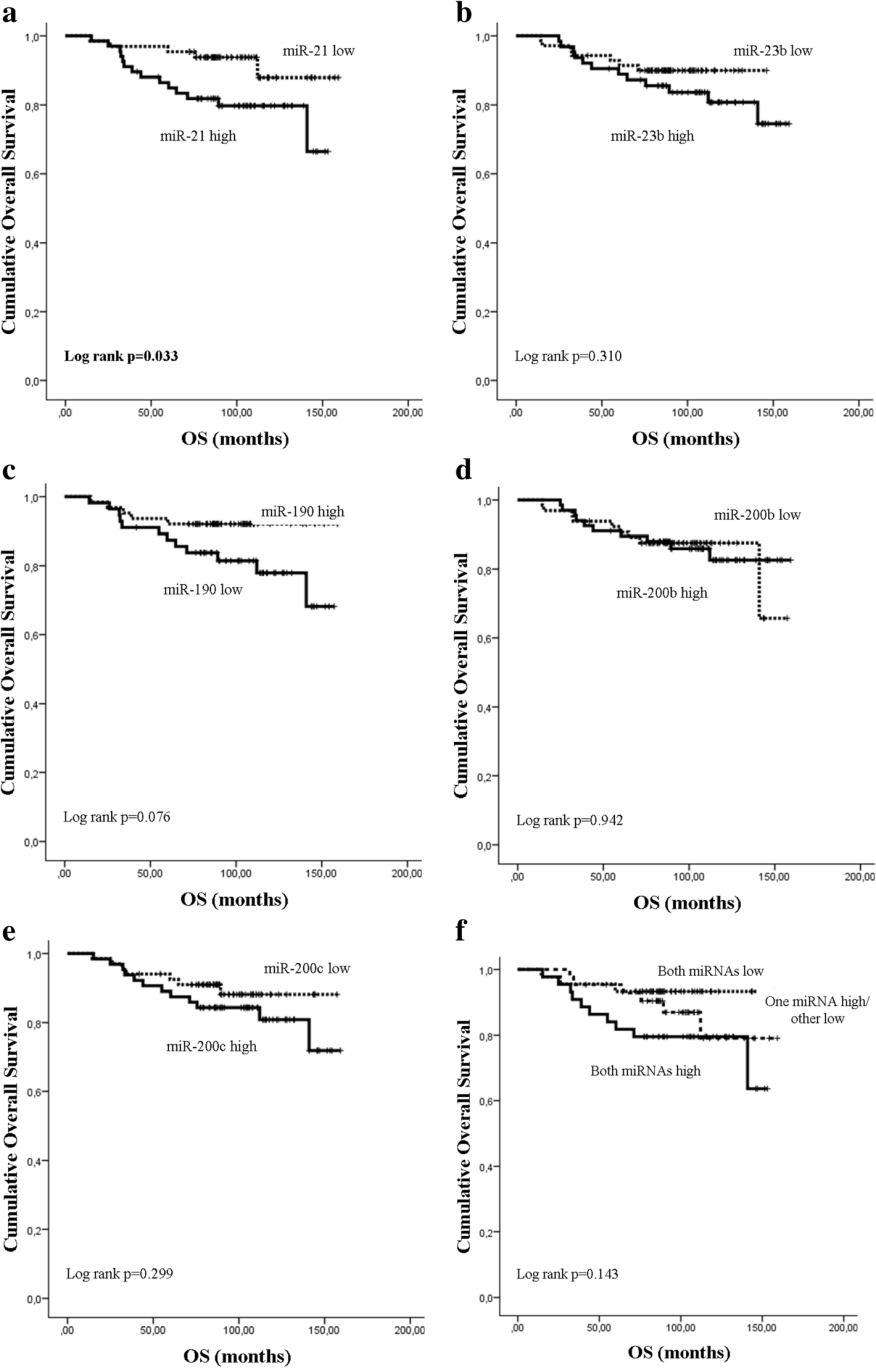
Kaplan-Meier analysis of overall survival (OS) according to the expression of circulating miRNAs and their combination. OS in patients with high or low expression of miR-21 (**a**), miR-23b (**b**), miR-190 (**c**), miR-200b (**d**), miR-200c (**e**) and the combination of miR-21 and miR-200c (**f**). Curves were compared using the log rank test. *P* values are shown

Median expression levels of miR-21, miR-23b and miR-200c were significantly higher (*p* < 0.001, *p* = 0.028 and *p* < 0.001, respectively) and median miR-190 expression was significantly lower (*p* = 0.013) in relapsed compared to non-relapsed patients (Fig. 2). No significant difference was observed in the median expression of miR-200b (*p* = 0.063) between the two groups. Subsequently, we evaluated the DFS (Fig. 3) and OS (Fig. 4) in patients classified into high and low expression groups, according to the median value of each miRNA. We found that patients with high miR-21 expression had significantly shorter DFS compared to patients with low expression (105.03 months versus not reached; *p* < 0.001) (Fig. 3a). Similarly, patients with high miR-200c expression had significantly shorter DFS compared to those with low miR-200c (105.03 vs not reached; *p* = 0.005) (Fig. 3e). Finally, patients with high expression of both miR-21 and miR-200c had shorter DFS compared to patients with only one miRNA high or with both low (81.37 vs 132.9 and not reached, respectively; *p* < 0.001) (Fig. 3f). No significant differences in DFS were found among patients with high or low expression of miR-23b, miR-190 or miR-200b (Fig. 3b-d). Median survival was not reached by patients with either high or low expression of any of the miRNAs evaluated (Fig. 4b-e). Nevertheless, only patients with high miR-21 had significantly shorter OS compared to those with low miR-21 (*p* = 0.033) (Fig. 4a).

To evaluate further the prognostic value of the circulating miRNAs, univariate and multivariate analyses were performed that included demographic and clinical variables and the expression levels of the five miRNAs (classified into high or low). Cox univariate analysis revealed that patients with infiltrated axillary lymph nodes and those with negative hormone receptor expression had significantly shorter DFS (*p* = 0.013 and *p* = 0.028, respectively) and OS (*p* = 0.044 and p = 0.01, respectively) (Table 4). High miR-21 and miR-200c expression levels were significantly associated with shorter DFS (*p* < 0.001 and *p* = 0.007, respectively) and only miR-21 high expression was associated with shorter OS (*p* = 0.042) (Table 4). Cox multivariate analysis revealed that the involvement of axillary lymph nodes and hormone receptor negativity were independent prognostic factors for shorter DFS (*p* = 0.019 and *p* = 0.012, respectively) and OS (*p* = 0.029 and *p* = 0.006, respectively) (Table 4). Furthermore, only high miR-21 and high miR-200c expression emerged as independent prognostic factors associated with shorter DFS (*p* = 0.003 and *p* = 0.037) (Table 4).

**Table 4:** Univariate and multivariate analysis for DFS and OS in patients with early breast cancer

	Univariate analysis			
	DFS		OS	
	HR (95% CI)	*p* value	HR (95% CI)	*p* value
Tumor size, T2–3 vs T1	1.48 (0.832–2.633)	0.177	1.584 (0.623–4.026)	0.334
Lymph nodes, pos vs neg	2.288 (1.192–4.391)	0.013	3.553 (1.034–12.206)	0.044
Histology grade, III vs I/II	1.650 (0.910–3.003)	0.099	1.276 (0.501–3.251)	0.609
ER, neg vs pos	1.318 (0.731–2.375)	0.359	2.120 (0.857–5.244)	0.104
PR, neg vs pos	2.003 (1.131–3.549)	0.017	2.323 (0.940–5.743)	0.068
ER/PR, neg vs at least one pos	2.010 (1.080–3.741)	0.028	3.324 (1.329–8.314)	0.01
Her2 pos vs neg	1.649 (0.771–3.525)	0.197	1.474 (0.429–5.065)	0.538
miR-21 high vs low	2.896 (1.556–5.390)	<0.001	2.884 (1.038–8.013)	0.042
miR-23b high vs low	1.624 (0.903–2.919)	0.105	1.630 (0.629–4.227)	0.315
miR-190 low vs high	1.342 (0.749–2.405)	0.322	2.511 (0.877–7.186)	0.086
miR-200b high vs low	1.460 (0.821–2.599)	0.198	1.034 (0.417–2.565)	0.942
miR-200c high vs low	2.287 (1.258–4.156)	0.007	1.637 (0.640–4.184)	0.304
Both miR-21/mir-200c high vs others	2.360 (1.346–4.135)	0.003	2.225 (0.902–5.489)	0.082
	Multivariate analysis			
	DFS		OS	
	HR (95% CI)	*p* value	HR (95% CI)	*p* value
Lymph nodes, pos vs neg	2.202 (1.138–4.260)	0.019	4.006 (1.151–13.935)	0.029
ER/PR, neg vs at least one pos	2.275 (1.202–4.305)	0.012	3.668 (1.457–9.233)	0.006
miR-21 high vs low	4.557 (1.685–12.869)	0.003	–	–
miR-200c high vs low	3.158 (1.074–9.288)	0.037		

*DFS* disease-free survival, *OS* overall survival, *pos* positive, *neg* negative, *ER* estrogen receptor, *PR* progesterone receptor, *HER2* human epidermal growth factor receptor 2

### miRNA expression according to the timing of recurrence

**Fig. 5:**
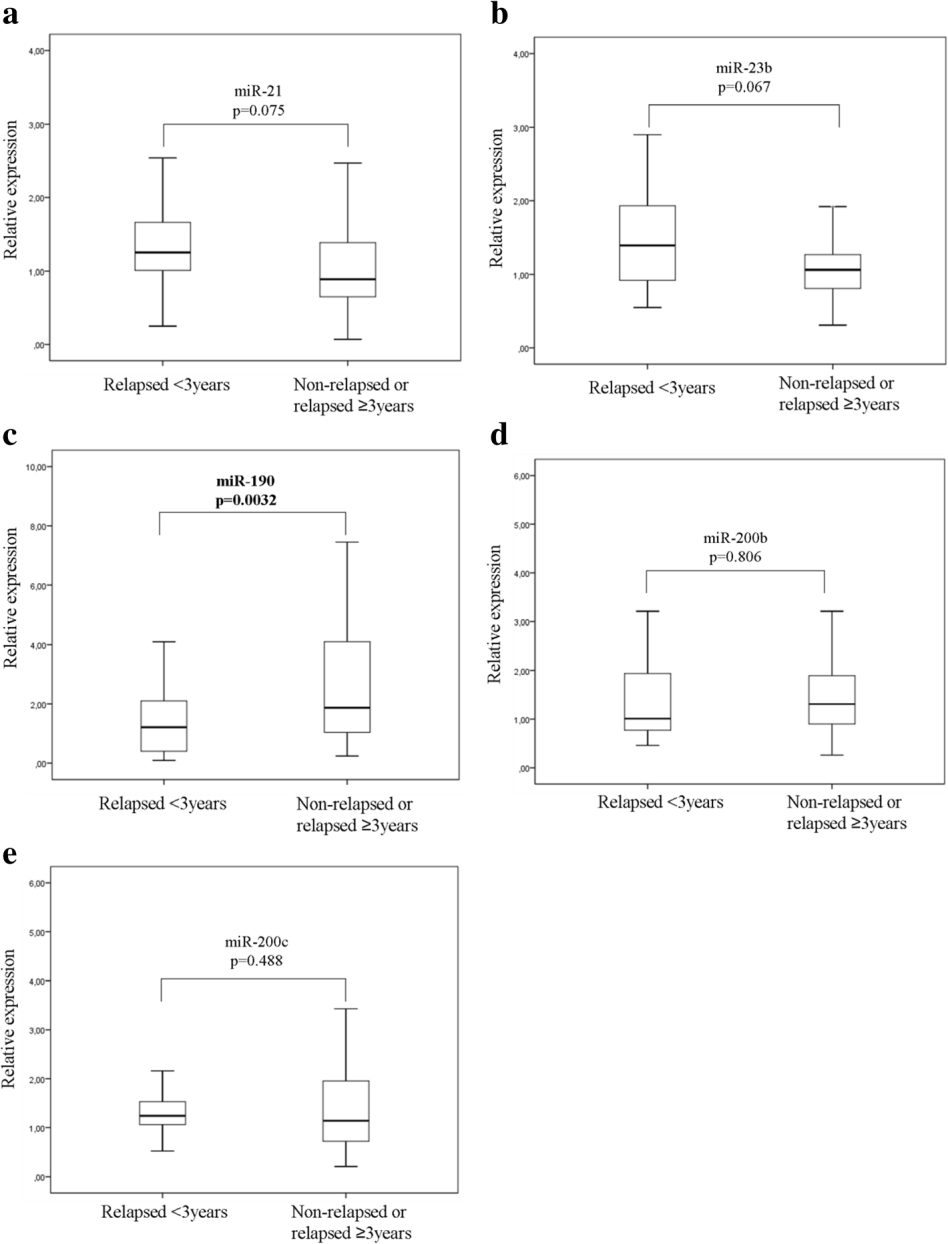
Differential expression of the five circulating miRNAs in patients with early relapse. Relative expression levels of miR-21 (**a**), miR-23 (**b**), miR-190 (**c**), miR-200b (**d**) and miR-200c (**e**) in plasma from patients that experienced early relapse (< 3 years) compared to those without early relapse. Statistically significant differences were determined using the Mann-Whitney test

**Fig. 6:**
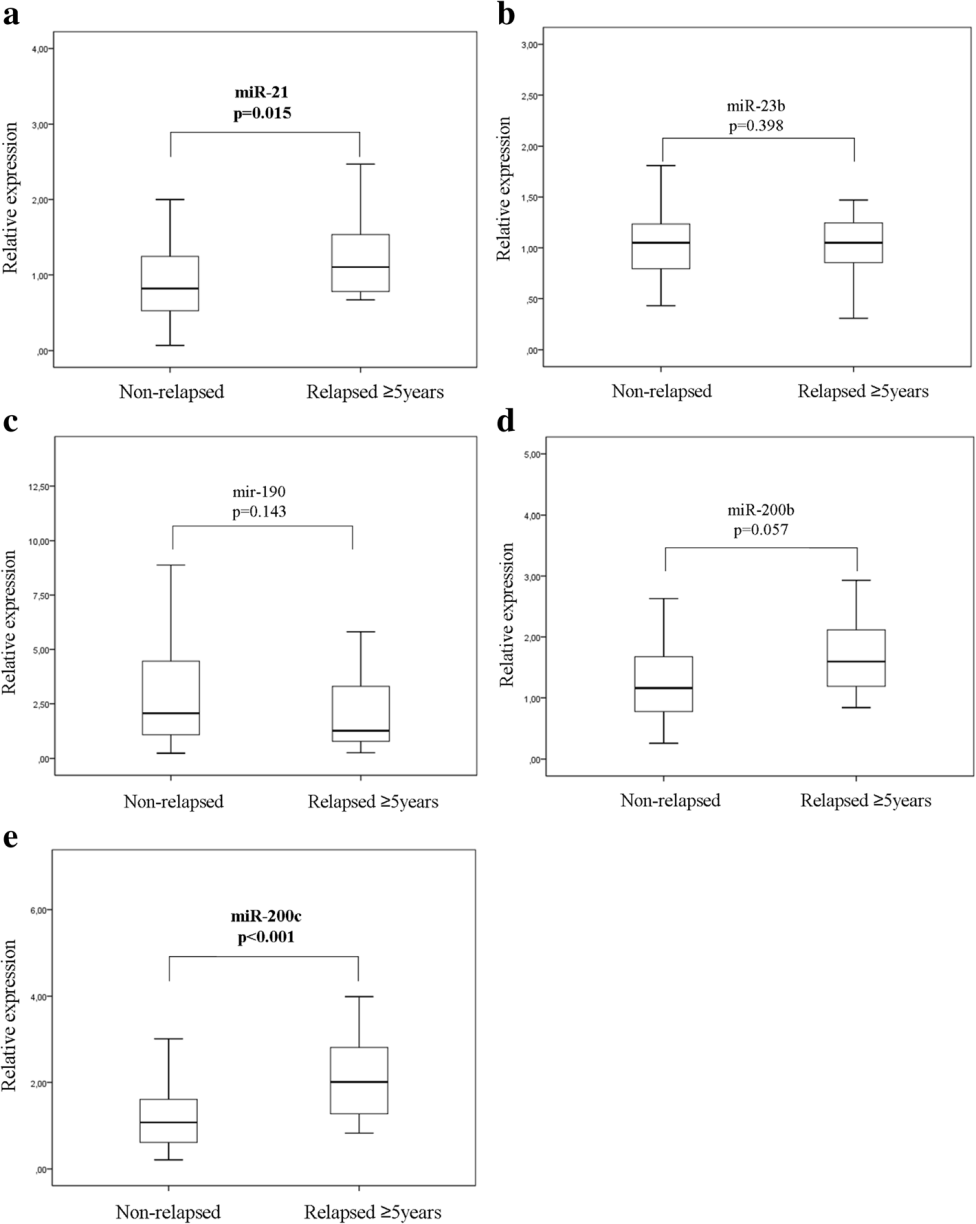
Differential expression of the five circulating miRNAs in patients with late relapse. Relative expression levels of miR-21 (**a**), miR-23 (**b**), miR-190 (**c**), miR-200b (**d**) and miR-200c (**e**) in plasma from patients who relapsed late (≥ 5 years) compared to patients without relapse during the follow-up. Statistically significant differences were determined using the Mann-Whitney test

We examined further whether the five circulating miRNAs are differentially expressed among patients classified into groups according to the timing of recurrence. For this purpose we compared miRNA expression levels in (i) patients who relapsed early compared to those who did not experience early relapse i.e. in patients who had recurrence within 3 years (*n* = 23) and those who either relapsed 3 or more years post-surgery or remained disease-free for the whole follow-up period (*n* = 110) and (ii) in patients with late relapse (at ≥ 5 years; *n* = 20) compared to those who remained disease-free during the whole follow-up period (*n* = 84). The Mann-Whitney test revealed that miR-190 expression levels were lower in patients with early relapse (*p* = 0.0032), whereas no differences were recorded for the remaining miRNAs (Fig. 5). Moreover, miR-21 and miR-200c expression was higher in patients with late relapse as compared to non-relapsed patients (*p* = 0.015 and *p* < 0.001, respectively; Fig. 6a and e).

### Combination of miRNA expression and clinicopathological characteristics in a relapse-predictive model

**Fig. 7:**
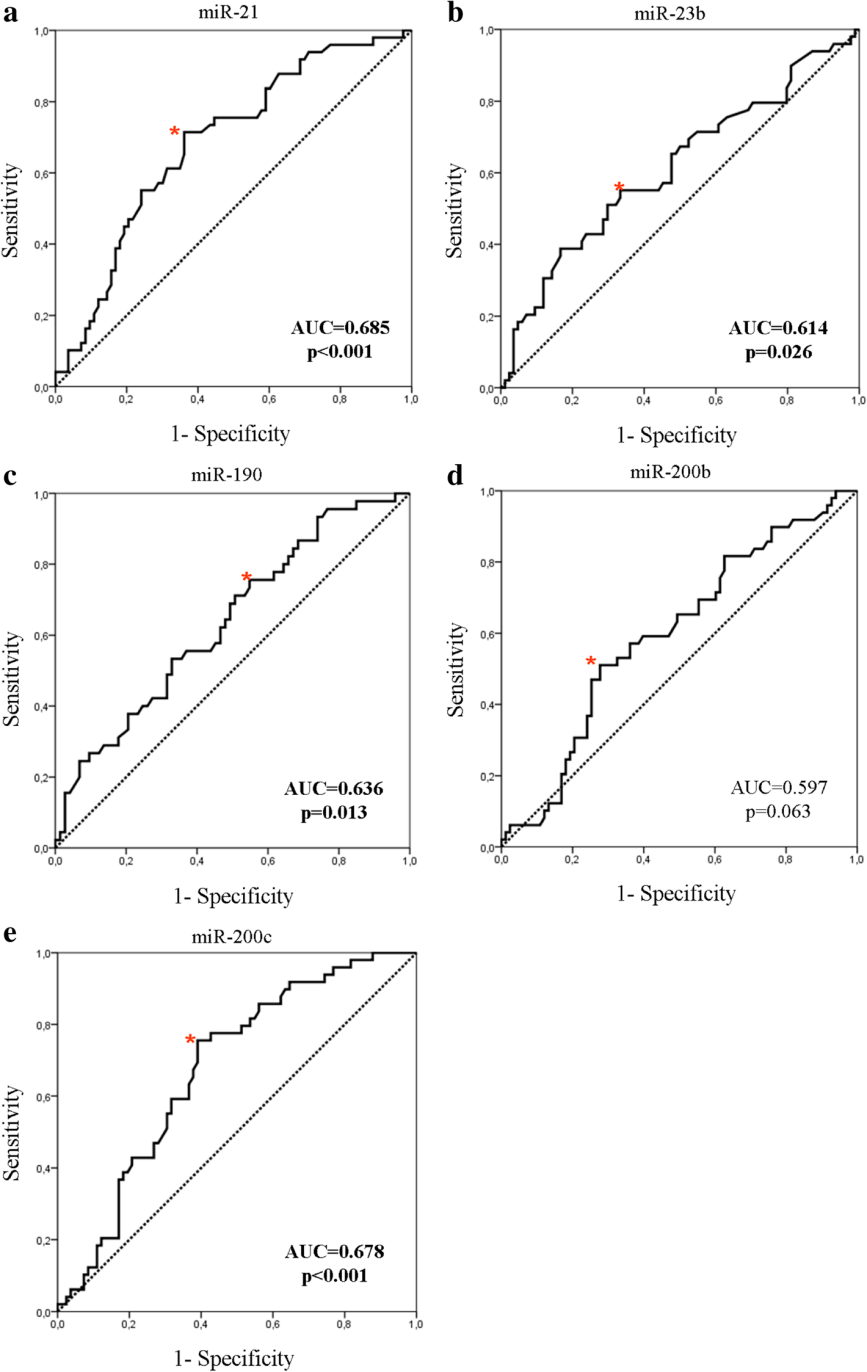
ROC curve analysis. Plasma miR-21 (**a**), miR-23 (**b**), miR-190 (**c**), miR-200b (**d**) and miR-200c (**e**) and their ability to discriminate between patients with relapse and those without relapse. Red asterisk indicates the highest Youden’s score index (sensitivity + specificity-1). AUC, area under the curve

**Fig. 8:**
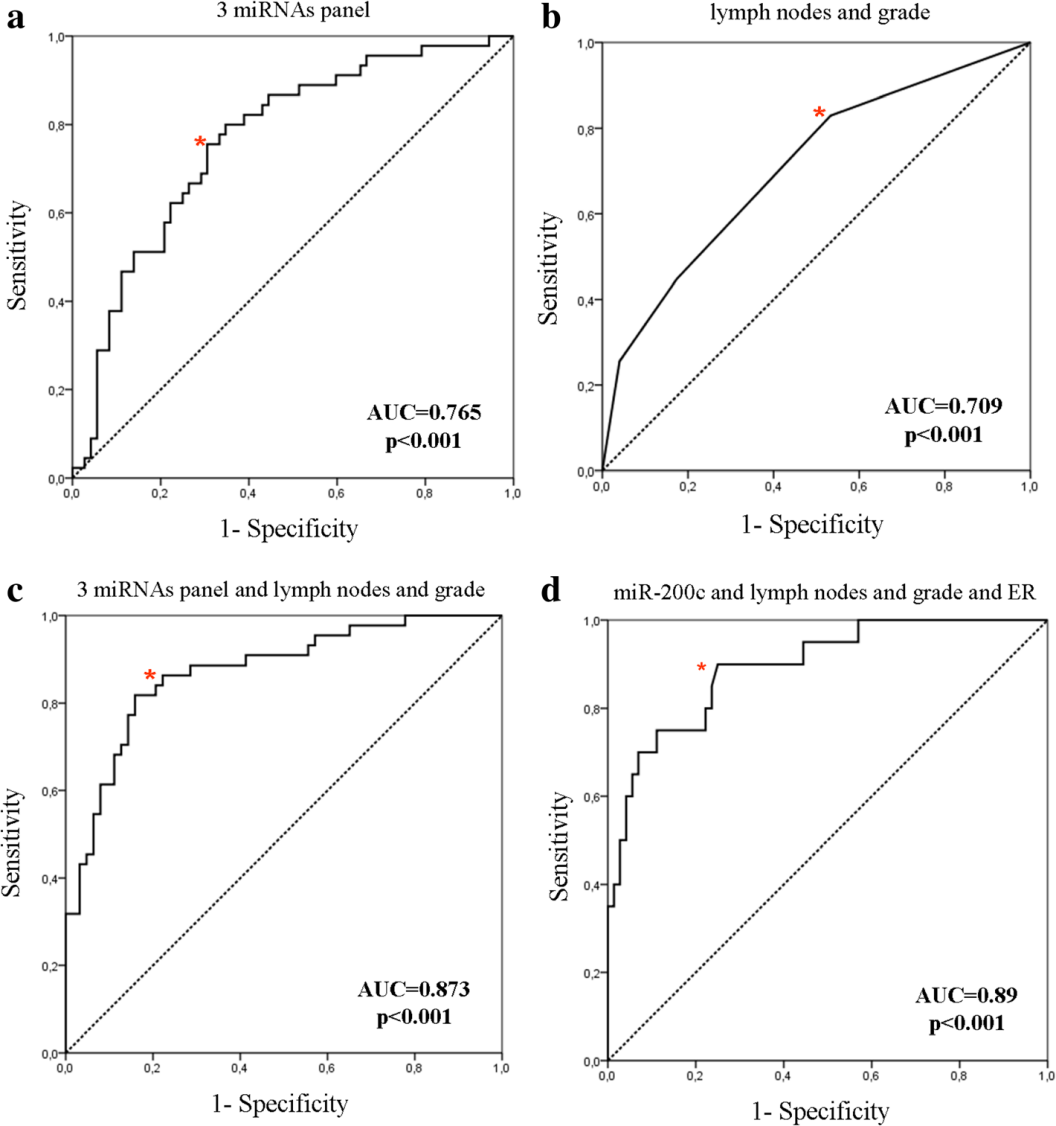
Combined ROC curve analysis. A three-miRNA panel (**a**) and clinicopathological parameters (**b**) alone or a three-miRNA panel in combination with clinicopathological parameters (**c**) and their ability to discriminate between patients with relapse and those without relapse and between patients with late relapse (≥ 5 years) compared to those without relapse (**d**). Red asterisk indicates the highest Youden’s score index (sensitivity + specificity-1)

Expression levels of various miRNAs were combined with clinicopathological characteristics in relapse-predicting models. We used binary logistic regression incorporating various combinations of miRNAs and used the corresponding ROC curves to determine the sensitivity and the specificity of plasma miRNA expression, to discriminate patients who subsequently had disease recurrence from non-relapsed patients (Fig. 7 and Table 5). When assessing single miRNAs, the ROC curves showed that the expression of miR-21 and miR-200c had the highest performance with an area under the ROC curve (AUC) of 0.685 (sensitivity 71.4%, specificity 63.9% (*p* < 0.001; 95% CI 0.592–0.777)) and AUC of 0.678 (sensitivity 75.5%, specificity 61% (*p* < 0.001; 95% CI 0.586–0.769)), respectively (Fig. 7a and e, respectively and Table 5). When assessing combinations of miRNAs, binary logistic regression analysis resulted in a pattern of three miRNAs (miR-21, miR-23b and miR-190) bearing the highest predictive accuracy. The AUC from ROC analysis of this combined model 0.765, with sensitivity 80% and specificity 65.3% (*p* < 0.001; 95% CI 0.673–0.850) (Fig. 8a and Table 5). Eventually, the combination of the three miRNAs with the currently used clinical prognostic parameters, axillary lymph node infiltration and tumor grade, resulted in superior discriminatory capability (AUC 0.873, sensitivity 89% and specificity 76.2% (*p* < 0.001; 95% CI 0.802–0.940)) compared to the expression of the three miRNAs alone or to the clinicopathological features alone (Fig. 8b, c and Table 5). Using the same procedure the combination of miR-200c expression, axillary lymph node infiltration, tumor grade and ER status resulted in an increased AUC of 0.890 with a sensitivity 75% and specificity 89% (*p* < 0.001; 95% CI 0.818–0.972) for the prediction of late disease relapse (Fig. 8d and Table 5). When the same model was fitted to predict early relapse, there were no differences in the discriminatory power when combining miRNAs with clinicopathological parameters.

**Table 5:** Performance of miRNAs and their combinations to predict relapse in patients with early breast cancer

Potential predictors	Cutoff value	Sensitivity (%)	Specificity (%)	AUC (95% CI)	*p*
Early breast cancer					
miR-21	0.98	71.4	63.9	0.685 (0.592–0.777)	< 0.001
miR-23b	1.35	38.8	83.3	0.614 (0.512–0.716)	0.029
miR-190	2.36	75.6	45.2	0.636 (0.534–0.738)	0.013
miR-200b	1.72	51.0	72.3	0.597 (0.498–0.696)	0.063
miR-200c	1.15	75.5	61.0	0.678 (0.586–0.769)	< 0.001
Three miRNAS (miR-21, miR-23b, miR-190)	0.39	80	65.3	0.765 (0.673–0.850)	< 0.001
Lymph nodes and grade	0.29	83	46.7	0.709 (0.614–0.804)	< 0.001
Three miRNAS plus lymph nodes and grade	0.41	89	76.2	0.873 (0.802–0.940)	< 0.001
Late relapse (≥ 5 years)					
miR-200c and lymph nodes, grade and ER status	0.42	75	89	0.89 (0.812–0.972)	< 0.001

*AUC* area under the receiver operating curve, ER estrogen receptor

The robustness of the predictive performance of our models was assessed through a cross-validation strategy. A 10-fold cross-validation with a 70–30 split (70% training data, 30% testing data) was implemented in R and applied on nine different feature combinations of miRNAs and clinicopathological features. Mean AUC values were calculated for each 10-fold cross-validation. The mean AUC was then compared to the AUC calculated from our initial regression analysis. There were no significant differences in the values of AUC in any variable combinations, indicating that the performance of these models is robust and can be generalized to independent datasets (Additional file 1: Table S1).

## Discussion

An important area in current breast cancer research is the identification of novel biomarkers for the prediction of outcome in patients with early disease. In the present study we investigated the predictive capacity of the dormancy and metastasis-related miR-21, miR-23b, miR-190, miR-200b and miR-200c when determined in the plasma of patients with early breast cancer. We found that miR-21, miR-23b, miR-190 and miR-200c, evaluated before the initiation of adjuvant therapy, were differentially expressed among patients who subsequently experienced disease recurrence, compared to patients who did not relapse. High expression of miR-21 and miR-200c was associated with shorter DFS compared to patients with low expression, whereas high miR-21 was also associated with shorter OS. Interestingly, miR-21, miR-23b, miR-190 and miR-200c discriminated patients who relapsed from non-relapsed patients. The combination of miR-21, miR-23b and miR-190 in ROC curve analyses had higher sensitivity and specificity compared to each miRNA alone; accuracy was further improved by adding lymph node infiltration and tumor grade to the panel of three miRNAs. Furthermore, the combination of miR-200c, lymph node infiltration, tumor grade and ER status predicted late relapse.

In breast cancer, clinically detectable metastases emerge after a period of dormancy and can last for varying and frequently prolonged periods of time. As miRNAs regulate tumor progression and metastasis we hypothesized that dormant tumors could be distinguished from faster-growing tumors by the differential expression of miRNAs [26]. We show for the first time that miR-190 expression was lower in patients with early relapse, suggesting a potential role for this miRNA in sustaining tumor dormancy in breast cancer. Indeed, miR-190 was among the most upregulated miRNAs in a dormancy-related miRNA signature [21]. miR-190 is involved in the regulation of the transforming growth factor (TGF)β pathway and in breast cancer TGFβ has been shown to promote bone and lung metastases [27, 28]. Thus, miR-190 could induce tumor dormancy through the modulation of TGFβ signaling [29].

Previous studies showed that miR-23b induced dormant phenotypes in a bone marrow, metastatic, human breast cancer cell line, induced cell cycle arrest in glioma cancer stem cells and suppressed glioma cell migration and invasion [20, 30, 31] . On the contrary, the miR-23b/27b/24 cluster correlated with increased metastatic potential in human breast cancer cell lines and was upregulated in lung metastases from breast cancer [32]. Moreover, high miR-23b/27b/24 expression was associated with poor outcome in breast cancer [33]. Our results demonstrate higher plasma miR-23b expression in patients who relapsed, indicating that it is more likely associated with the development of metastases in breast cancer. Interestingly, the mature sequence of miR-23a differs by just one nucleotide in comparison to its paralog miR-23b, therefore they could share the same putative target genes and similar biological functions. However, there are reports showing distinct function between miR-23a and miR-23b and in contrast to miR-23b, we detected no variations in miR-23a expression levels among the different patient cohorts [34, 35].

Various preclinical studies have established that miR-21 is involved in tumor growth, invasion and migration, extracellular matrix modification and survival [36]. In primary breast cancer, miR-21 expression is associated with tumor progression, advanced clinical stage, lymph node metastasis and poor patient outcome [37, 38]. In support of the tumor-promoting role of miR-21, serum miR-21 distinguishes patients with breast cancer from healthy controls and patients with distant metastasis from those with locoregional disease, and it is associated with poor prognosis in breast cancer [36, 39, 40]. Accordingly, we show that high circulating miR-21 discriminated between patients with early breast cancer who relapsed and those who remained disease-free and specifically, high expression was associated with late relapse. Importantly, patients with high plasma miR-21 expression levels had worse DFS and OS compared to patients with low expression, whereas high miR-21 also emerged as an independent predictive factor for shorter DFS (*p* = 0.003). Iorio et al., demonstrated that the TGFβ gene was a target for miR-21 and Yan et al. showed that TGFβ1 and the receptor TGFβR2 were identified among the putative target genes of miR-21 [37, 38]. These data suggest that the tumor promoting effects of miR-21 in breast cancer, could be exerted through the regulation of TGFβ signaling.

The miR-200 family (miR-200a, miR-200b, miR-200c, miR-141 and miR-429) has opposing roles in the regulation of EMT and metastasis [41]. On one hand, they negatively regulate the E-cadherin transcriptional repressors ZEB1/2 preventing EMT and on the other, they have been associated with global shifts in gene expression which promote metastatic colonization in breast cancer mouse models [17, 42]. Conflicting results have been also reported on the clinical relevance of miR-200 family members in breast cancer [43, 44]. By adopting a global profiling approach, Madhavan et al. showed that miR-200b and miR-200c were among the panel of six miRNAs with significantly increased expression in patients with early breast cancer who developed metastases [45]. Our results also support the association between the plasma miR-200 family and metastatic progression in breast cancer. Importantly, high miR-200c was associated with late relapse and emerged as an independent prognostic factor for worse DFS (*p* = 0.037).

ROC curve analysis confirmed the value of the plasma miRNAs in the prediction of disease recurrence in breast cancer. The combination of miR-21, miR-23b and miR-190 had higher accuracy compared to each miRNA alone. Moreover, the addition of common clinicopathological prognostic factors further improved the discriminatory capability of the three miRNAs. These results provide novel opportunities for breast cancer therapeutics employing the aforementioned miRNAs in a combinatorial miRNA approach [46]. From a network analysis perspective, further insights might be achieved through the incorporation of information on the expression of the protein-coding mRNA associated to the involved miRNA. The formulation of a model of intervention efficiency based on a combination of miRNA, their gene targets and associated pathways would thus provide complementary information orthogonal to the one obtained from pathological characteristics.

In breast cancer, late relapses are common and impose considerable concern among disease-free patients, and there are no accurate tools to identify patients at risk. Importantly, in our study miR-200c expression combined with the clinical information on axillary lymph node status, tumor grade and ER status yielded an AUC of 0.89 with sensitivity of 75% and specificity of 89% for the prediction of late relapse (*p* < 0.001).

Our study is among the first to demonstrate the potential of metastasis-promoting miRNAs to serve as circulating predictive markers in early breast cancer. Importantly, (a) this patient cohort had long-term follow up, (b) plasma samples and clinical information were obtained prospectively, (c) the prediction of relapse was possible years before metastasis emerged and (d) circulating miRNAs added independent predictive value to common clinicopathological parameters. Furthermore, we considered pre-analytical and analytical parameters very carefully, taking into account the variables that could lead to bias in miRNA quantification [22, 47].

Limitations of our study include that results are derived from the analysis of a relatively small group of patients and lack validation in an independent cohort. However, by performing cross-validation analysis of our data [48], the predictive performance of the aforementioned miRNAs was confirmed, therefore it could probably be verified in an independent dataset. Nevertheless, our results should be viewed as preliminary and warrant prospective validation in a larger cohort of patients with early disease.

## Conclusions

Our results suggest that dormancy and metastasis-related miRNAs are differentially expressed in plasma in patients with early breast cancer who experience disease recurrence and in those that will remain disease-free. The identified miRNAs might be of potential use in the development of a multimarker blood-based test to complement and improve prognostication based on clinicopathological characteristics. Furthermore, these results imply that circulating miRNAs could serve as novel surrogate markers for the presence of occult micro metastatic disease and for increased risk of recurrence in early breast cancer. Finally, they provide potential insights into the procedures and pathways involved in the regulation of dormancy and metastasis in breast cancer.

## Additional file

Additional file 1: **Table S1.** Tenfold cross-validation results of nine different sets of combinations of predictor variables. (DOCX 13 kb)

## Abbreviations

AUC: Area under the curve
cDNA: Complementary DNA
Ct: Cycle threshold
DFS: Disease-free survival
EMT: Epithelial-mesenchymal transition
ER: Estrogen receptor
HER2: Human epidermal growth factor receptor 2
miR: Micro-RNA
OS: Overall survival
PR: Progesterone receptor
ROC: Receiver operating characteristic
TGF: Transforming growth factor

## References

[1] Brewster AM (2008). Residual risk of breast cancer recurrence 5 years after adjuvant therapy. J Natl Cancer Inst.

[2] Colleoni M (2016). Annual hazard rates of recurrence for breast cancer during 24 years of follow-up: results from the international breast cancer study group trials I to V. J Clin Oncol.

[3] Davies C (2013). Long-term effects of continuing adjuvant tamoxifen to 10 years versus stopping at 5 years after diagnosis of oestrogen receptor-positive breast cancer: ATLAS*,* a randomised trial. Lancet.

[4] Aguirre-Ghiso JA (2007). Models, mechanisms and clinical evidence for cancer dormancy. Nat Rev Cancer.

[5] Pan H (2017). 20-Year risks of breast-cancer recurrence after stopping endocrine therapy at 5 years. N Engl J Med.

[6] Sestak I, Cuzick J (2015). Markers for the identification of late breast cancer recurrence. Breast Cancer Res.

[7] Chen N (2013). Incorporate gene signature profiling into routine molecular testing. Appl Transl Genom.

[8] Bedard PL (2013). Tumour heterogeneity in the clinic. Nature.

[9] Bartel DP (2009). MicroRNAs: target recognition and regulatory functions. Cell.

[10] Hayes J, Peruzzi PP, Lawler S (2014). MicroRNAs in cancer: biomarkers, functions and therapy. Trends Mol Med.

[11] Peng Y, Croce CM (2016). The role of MicroRNAs in human cancer. Signal Transduction And Targeted Therapy.

[12] Schwarzenbach H (2014). Clinical relevance of circulating cell-free microRNAs in cancer. Nat Rev Clin Oncol.

[13] Mitchell PS (2008). Circulating microRNAs as stable blood-based markers for cancer detection. Proc Natl Acad Sci U S A.

[14] Turchinovich A (2011). Characterization of extracellular circulating microRNA. Nucleic Acids Res.

[15] Cheng Q (2014). A signature of epithelial-mesenchymal plasticity and stromal activation in primary tumor modulates late recurrence in breast cancer independent of disease subtype. Breast Cancer Res.

[16] De Cock JM (2016). Inflammation triggers Zeb1-dependent escape from tumor latency. Cancer Res.

[17] Gregory PA (2008). The miR-200 family and miR-205 regulate epithelial to mesenchymal transition by targeting ZEB1 and SIP1. Nat Cell Biol.

[18] Dykxhoorn DM (2009). miR-200 enhances mouse breast cancer cell colonization to form distant metastases. PLoS One.

[19] Zhu S (2008). MicroRNA-21 targets tumor suppressor genes in invasion and metastasis. Cell Res.

[20] Ono M (2014). Exosomes from bone marrow mesenchymal stem cells contain a microRNA that promotes dormancy in metastatic breast cancer cells. Sci Signal.

[21] Almog N (2013). Transcriptional changes induced by the tumor dormancy-associated microRNA-190. Transcription.

[22] Schwarzenbach H (2015). Data normalization strategies for MicroRNA quantification. Clin Chem.

[23] Schmittgen TD, Livak KJ (2008). Analyzing real-time PCR data by the comparative C(T) method. Nat Protoc.

[24] Blondal T (2013). Assessing sample and miRNA profile quality in serum and plasma or other biofluids. Methods.

[25] McShane LM (2005). Reporting recommendations for tumor marker prognostic studies. J Clin Oncol.

[26] Almog N (2012). Consensus micro RNAs governing the switch of dormant tumors to the fast-growing angiogenic phenotype. PLoS One.

[27] Gennarino VA (2012). Identification of microRNA-regulated gene networks by expression analysis of target genes. Genome Res.

[28] Drabsch Y, ten Dijke P (2011). TGF-beta signaling in breast cancer cell invasion and bone metastasis. J Mammary Gland Biol Neoplasia.

[29] Bragado P (2013). TGF-beta2 dictates disseminated tumour cell fate in target organs through TGF-beta-RIII and p38alpha/beta signalling. Nat Cell Biol.

[30] Geng J (2012). Methylation mediated silencing of miR-23b expression and its role in glioma stem cells. Neurosci Lett.

[31] Loftus JC (2012). miRNA expression profiling in migrating glioblastoma cells: regulation of cell migration and invasion by miR-23b via targeting of Pyk2. PLoS One.

[32] Ell B (2014). The microRNA-23b/27b/24 cluster promotes breast cancer lung metastasis by targeting metastasis-suppressive gene prosaposin. J Biol Chem.

[33] Jin L (2013). Prooncogenic factors miR-23b and miR-27b are regulated by Her2/Neu, EGF, and TNF-alpha in breast cancer. Cancer Res.

[34] Lin R (2014). Targeting miR-23a in CD8+ cytotoxic T lymphocytes prevents tumor-dependent immunosuppression. J Clin Invest.

[35] Li J (2016). The poly-cistronic miR-23-27-24 complexes target endothelial cell junctions: differential functional and molecular effects of miR-23a and miR-23b. Mol Ther Nucleic Acids.

[36] Aleckovic M, Kang Y (2015). Regulation of cancer metastasis by cell-free miRNAs. Biochim Biophys Acta.

[37] Iorio MV (2005). MicroRNA gene expression deregulation in human breast cancer. Cancer Res.

[38] Yan LX (2008). MicroRNA miR-21 overexpression in human breast cancer is associated with advanced clinical stage, lymph node metastasis and patient poor prognosis. RNA.

[39] Wang G (2015). Quantitative measurement of serum microRNA-21 expression in relation to breast cancer metastasis in Chinese females. Ann Lab Med.

[40] Muller V (2014). Changes in serum levels of miR-21, miR-210, and miR-373 in HER2-positive breast cancer patients undergoing neoadjuvant therapy: a translational research project within the Geparquinto trial. Breast Cancer Res Treat.

[41] Humphries B, Yang C (2015). The microRNA-200 family: small molecules with novel roles in cancer development, progression and therapy. Oncotarget.

[42] Korpal M (2011). Direct targeting of Sec23a by miR-200s influences cancer cell secretome and promotes metastatic colonization. Nat Med.

[43] Song (2015). C, et al. miR-200c inhibits breast cancer proliferation by targeting KRAS. Oncotarget.

[44] Antolin S (2015). Circulating miR-200c and miR-141 and outcomes in patients with breast cancer. BMC Cancer.

[45] Madhavan D (2012). Circulating miRNAs as surrogate markers for circulating tumor cells and prognostic markers in metastatic breast cancer. Clin Cancer Res.

[46] Kasinski AL (2015). A combinatorial microRNA therapeutics approach to suppressing non-small cell lung cancer. Oncogene.

[47] McDonald JS (2011). Analysis of circulating microRNA: preanalytical and analytical challenges. Clin Chem.

[48] Machiela MJ (2011). Evaluation of polygenic risk scores for predicting breast and prostate cancer risk. Genet Epidemiol.

