# Correction to: Circulating microRNAs in the early prediction of disease recurrence in primary breast cancer

**DOI:** 10.1186/s13058-022-01544-w

**Published:** 2022-07-22

**Authors:** Chara Papadaki, Michalis Stratigos, Georgios Markakis, Maria Spiliotaki, Georgios Mastrostamatis, Christoforos Nikolaou, Dimitrios Mavroudis, Sofia Agelaki

**Affiliations:** 1 Laboratory of Translational Oncology, School of Medicine, University of Crete, Heraklion, 71003 Heraklion, Crete, Greece; 2 Department of Medical Oncology, University General Hospital of Heraklion, 1352 PO BOX, 711 10 Heraklion, Crete, Greece; 3 Department of Agricultural, Technological Education Institute of Heraklion, 72100 Heraklion, Crete, Greece; 4 Computational Genomics Group, Department of Biology, University of Crete, 70013 Heraklion, Greece; 5 Institute of Molecular Biology and Biotechnology, Foundation for Research and Technology, 70013 Heraklion, Crete, Greece

## Correction to: Breast Cancer Research (2018) 20:72 10.1186/s13058-018-1001-3

Following publication of the original article [1], the authors identified an error in Dr. Michalis Stratigos’s affiliation.

It currently reads:

^2^Department of Medical Oncology, University General Hospital of Heraklion, 1352 PO BOX, 711 10 Heraklion, Crete, Greece

It should read:

^1^Laboratory of Translational Oncology, School of Medicine, University of Crete, Heraklion, 71003 Heraklion, Crete, Greece

^2^Department of Medical Oncology, University General Hospital of Heraklion, 1352 PO BOX, 711 10 Heraklion, Crete, Greece

The original article [1] has been corrected.
