# Supplementary material for: Circulating microRNAs in the early prediction of disease recurrence in primary breast cancer
Source: Breast Cancer Res. 2018 Jul 11;20:72. doi: 10.1186/s13058-018-1001-3 (PMC6042266; doi:10.1186/s13058-018-1001-3)
Supplement: Supplementary file 1 — Table S1. Tenfold cross-validation results of nine different sets of combinations of predictor variables. (DOCX 13 kb) [file 13058_2018_1001_MOESM1_ESM.docx]

**Additional file Table S1.** Ten-fold cross-validation results of nine different sets of combinations of predictor variables.

| **Potential Predictors** | **AUC (complete dataset)** | **Mean AUC**  **(10-fold cross-validation)** | **Mean p-value**  **(10-fold cross-validation)** |
| --- | --- | --- | --- |
| **Early breast cancer** |  |  |  |
| miR-21 | 0.685 | 0.679 | 0.0027 |
| miR-23b | 0.614 | 0.605 | 0.082 |
| miR190 | 0.636 | 0.628 | 0.034 |
| miR200b | 0.597 | 0.588 | 0.088 |
| miR200c | 0.678 | 0.676 | 0.0045 |
| 3 miRNAs (miR21, miR23b, miR190) | 0.765 | 0.749 | <0.001 |
| Lymph nodes and grade | 0.709 | 0.695 | 0.0017 |
| 3 miRNAs plus lymph nodes and grade | 0.873 | 0.849 | <0.001 |
| **Late Relapse** |  |  |  |
| miR-200c plus lymph nodes, grade and ER status | 0.89 | 0.876 | <0.001 |
